# Ethical Issues in Addressing Social Media Posts About Suicidal Intentions During an Online Study Among Youth: Case Study

**DOI:** 10.2196/mental.8971

**Published:** 2018-05-03

**Authors:** Sean D Young, Renee Garett

**Affiliations:** ^1^ University of California Institute for Prediction Technology Department of Family Medicine University of California, Los Angeles Los Angeles, CA United States; ^2^ ElevateU Los Angeles, CA United States

**Keywords:** suicide, social media, undergraduates

## Abstract

Due to the popularity of social media, researchers are increasingly conducting studies that monitor and analyze people’s health-related social media conversations. Because social media users can post about any topic at any time, no known best ethical practices exist as to whether and how to monitor participants’ posts for safety-related issues that might be unrelated to the study, such as expressions of suicidal intentions. This is a case study during a social media-based study on sleep and activity among freshman undergraduate students, where we by chance noticed that a student was using social media to express suicidal intentions. Although we connected the student to student psychological services in order to receive treatment, we encountered a number of barriers that initially prevented this from occurring, such as institutional review board and regulatory practices related to lack of experience with these newer types of studies. We discuss the implications of this experience for future research.

## Introduction

Severe depression and suicide are common public health concerns among college aged young adults. Approximately 15% of college undergraduates experience a depressive disorder, and suicide is a leading cause of death for university students in the United States [1-3]. The percentage of students with severe psychological issues seeking help at university centers increased from 16% in 2000 to 44% in 2010, with only 13% of suicides being from past clients of student counseling centers [4,5]. Identifying ways to monitor student psychological health, increase referral to psychological services, and reduce suicide is a top public health concern.

Over the past decade adolescents have been using social media at an increasing rate, with more than 94% of youth using some form of social media [6,7]. Recently, studies have shown that social media can be used to monitor psychological issues, such as depression [8] and suicide [9]. These can be monitored on social media by screening for key words that social media users discuss online [10-12] and emotions expressed in their posts [13].

While conducting a study using social media to monitor the Twitter activity and general health and wellness among 197 freshmen undergraduates [14,15], we encountered an unexpected situation. Although our primary study aim was to examine the relationship between social media and general health such as sleep and activity, our method of social media tracking uncovered one student who was expressing suicidal thoughts on Twitter. Institutional Review Board (IRB) protocols and consent forms often have a predetermined process in place designed to reduce risk and address potential mentions of suicide that might occur among participants during the course of the study. These suicide protocols are typically created based on collaboration between the IRB, which is experienced with these issues, and the study researchers, who are experienced with the type of research being conducted. However, due to the novelty and lack of studies using social media as a tool for monitoring health behavior, lack of suicide-related issues that have been documented in social media monitoring studies, and the (presumably) healthy student population we thought were studying, no suicide-related protocol was included in our study.

This case study describes the incident and the process we undertook to address the situation of encountering unexpected suicide-related posts from a student. We explore the implications of our experience and raise ethical questions to facilitate a conversation on how to address these concerns in the future.

## Methods

The study protocol was approved by the University of California, Los Angeles IRB. Participants (N=197, incoming freshman undergraduates) completed in-person consent to join a 3-month study to assess their stress, sleep, and social media patterns during their first quarter. We recruited students from Facebook advertisements posted on the freshman class of the 2019 page and in-person on campus. Recruitment occurred from mid-September through mid-October 2015. To qualify for the study, students had to be freshmen or first-year transfers, aged 18 to 21 years, in their first quarter, active Twitter users (>3 posts per week), and willing to allow us to follow them on Twitter.

Students completed an initial demographic survey; weekly surveys on stress, sleep, and emotions; and a final survey including the Patient Health Questionnaire. They received $5 worth of online gift cards for completing each survey. Participants received an additional $5 if they completed all 4 weekly surveys in a given month. Students who completed all weekly surveys could receive a total of $75. We collected students’ Twitter activity using the Twitter application program interface.

## Results

To analyze the Twitter data collected, we identified conversational tweets and created a list of the top 3 people most frequently written to by each student. During the data analysis, we identified 1 student who had tried numerous times to contact members of a popular teenage band rather than trying to tweet at other noncelebrity Twitter users as their peers did. We decided to further review the social media content of this student to determine differences between this student and others. During the course of this exploration, we discovered 6 tweets expressing suicidal ideation and severe depression.

These statements appeared to be credible and concerning. In consultation among our staff, including a licensed clinical social worker, along with the IRB, we determined that without a formal protocol on how to address this situation, we should immediately contact student psychological services to notify them of the incident and attempt to route the student to care with them. Upon contacting student psychological services, we discovered that they were unable to verify the student and veracity of the student’s intentions because university policy prohibited their staff from viewing students’ social media activity. Additionally, the student used a pseudonym on Twitter and in other forms filled out for the study. Due to this, staff members at psychological services were initially unable to identify the student in their database, causing delays in contacting the student. We later referenced the original consent sheet to discover the student’s legal name.

## Discussion

To our knowledge, only 1 study has explored expressions of suicidal ideation among social media users, and it explored how to theoretically mine social media data for suicidal content [16]. As social media becomes a more widely used tool in research, we believe it is essential for researchers using social media for monitoring to craft a plan before beginning the study to address identification of clinical issues in social media such as suicidal intentions. Although the American Psychological Association and American Counseling Association have ethical guidelines for integrating telehealth technologies into clinical practice and for health communications, this case study suggests a greater need to develop specific guidelines on how to respond to clinical issues from social media posts as part of a research study.

It is important to note that our research was not designed to study suicide or to directly study clinical health of students but rather to study and monitor general health behaviors such as sleep. Future researchers using social media as a tool for monitoring or interventions may therefore encounter similar situations where participants post about suicide or clinical issues even if the primary goal of the study is focused on issues unrelated to clinical care. This study therefore raises ethical questions on considerations researchers and ethical review boards should have when reviewing studies related to social media and online posting. For example, should a consent form include information that researchers might intervene if they detect clinical emergencies? Should researchers be required to act on this information? Are researchers required to conduct periodic or routine monitoring just because they have access to these data? Should researchers take steps to remind study participants who are publicly expressing clinical issues that they are in a study and being monitored? More broadly, when monitoring publicly available information on social media, in what situations should information be reported to IRBs, clinical experts, or police? These are just some of the many questions arising from this growing area of research.

We learned 3 additional lessons from this experience. First, we were fortunate to have a clinical expert assisting with the study in order to gain clinical expertise on how to address the issue immediately. It may be advisable that studies on social media monitoring have a clinical expert available to address emergency situations if the IRB does not have the appropriate clinical staff. Second, we encountered delays because student psychological services was unable to view the student’s social media profile to verify the student and assess the validity of their suicidal intentions. 

**Figure 1:**
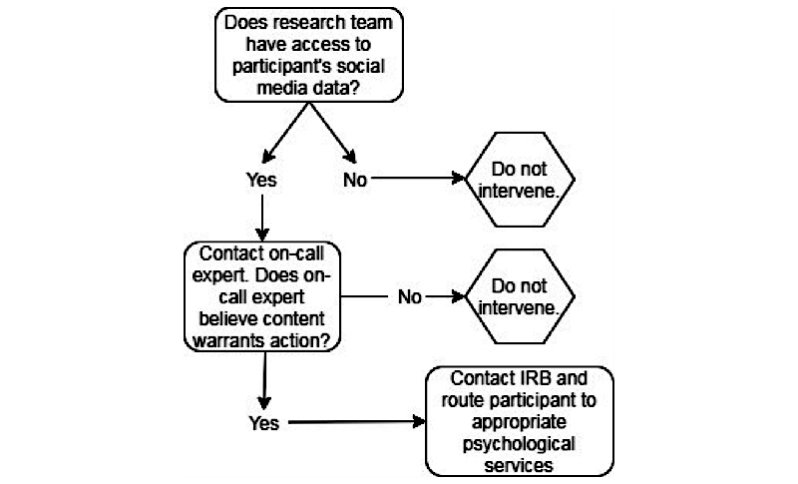
Potential protocol for addressing suicidal intentions in social media studies.

Although it is understandable that psychological services should not view student social media posts in order to maintain confidentiality and privacy, a plan should be in place to address this issue.

As social media is often publicly available, viewing public social media posts would be similar to overhearing a student talking on the street. Additional discussion on this topic could help to identify how and when participants in social media-based research studies receive emergency clinical care. Finally, our results have led us to believe that at minimum, all research studies that seek to monitor social media content from individuals, whether or not the topic is related to mental health, should include a provision for how to address potential suicidal content that may arise.

While we do not have a finalized protocol in place to address study participants expressing suicidal intentions, we describe a working draft of a potential protocol for future studies of this nature below and outline the protocol in Figure 1.

Researchers look at social media for various reasons including advertising for recruitment, monitoring, and intervention. If a person is actively enrolled in a study for monitoring or intervention and has gone through the consenting process, the study team should have a protocol in place to address suicidal thoughts or intentions expressed on social media.

If a person in the study is expressing suicidal thoughts or intentions on social media, it should be the responsibility of the research team to have a certified clinician on call to assess the social media posts and determine if action is warranted. If the posts are deemed dangerous or indicative of severe mental distress, the clinician should contact the IRB and reach out to the participant if needed to provide a referral to appropriate services. It should be made clear in the consent form that participants are agreeing to being contacted by this clinician if they express suicidal thoughts or intentions during the duration of the study.

We suggest ways to integrate our findings into future social media-based research protocols. First, researchers should have a process in place for how to address the risk of participants posting suicidal and clinical information in social media-based studies. Second, studies on this topic should have a strict set of procedures about whom to contact and what information to share with outside agencies that deal with psychological concerns that may present themselves during the course of the study. Although this is standard in many ethical protocols, there is limited discussion on whether and how to do this in social media-based research. Third, during recruitment and consent, effort should be taken to gain accurate contact information for participants, should the need arise to contact them.

Social media has become an increasingly common tool in research. Because people commonly use social media to publicly communicate their thoughts and behaviors, including those of a clinical nature, participants in social media-based studies may disclose personal clinical risks, such as suicidal intentions. As these types of incidents increase in prevalence along with social media-based research, it is imperative that researchers identify the ethical questions and frameworks that can address these issues.

## Abbreviations

IRB: institutional review board

## References

[1] Eisenberg D, Gollust SE, Golberstein E, Hefner JL (2007). Prevalence and correlates of depression, anxiety, and suicidality among university students. Am J Orthopsychiatry.

[2] Schwartz AJ (2006). College student suicide in the United States: 1990-1991 through 2003-2004. J Am Coll Health.

[3] Suicide Prevention Resource Center (2014). Suicide among college and university students in the United States.

[4] Eiser A (2011). The crisis on campus.

[5] Gallagher R, Taylor R (2010). National survey of counseling center directors.

[6] Perrin A (2015). Social media usage 2005-2015.

[7] (2012). US Department of Health and Human Services.

[8] De Choudury M, Counts S, Horvitz E, Gamon M Predicting depression via social media.

[9] Won H, Myung W, Song G, Lee W, Kim J, Carroll BJ, Kim DK (2013). Predicting national suicide numbers with social media data. PLoS One.

[10] O'Dea B, Wan S, Batterham P, Calear A, Paris C, Christensen H (2015). Detecting suicidality on Twitter. Internet Interv.

[11] Young Sean D, Rivers Caitlin, Lewis Bryan (2014). Methods of using real-time social media technologies for detection and remote monitoring of HIV outcomes. Prev Med.

[12] Young Sean D, Yu Wenchao, Wang Wei (2017). Toward Automating HIV Identification: Machine Learning for Rapid Identification of HIV-Related Social Media Data. J Acquir Immune Defic Syndr.

[13] Burnap P, Colombo W, Scourfield J (2015). Machine classification and analysis of suicide-related communication on Twitter.

[14] Liu Sam, Zhu Miaoqi, Yu Dong Jin, Rasin Alexander, Young Sean D (2017). Using Real-Time Social Media Technologies to Monitor Levels of Perceived Stress and Emotional State in College Students: A Web-Based Questionnaire Study. JMIR Ment Health.

[15] Garett Renee, Liu Sam, Young Sean D (2017). A longitudinal analysis of stress among incoming college freshmen. J Am Coll Health.

[16] Abboute A, Boudjeriou Y, Entringer G, Azé J, Bringay S, Poncelet P, Métais E, Roche M, Teisseire M (2014). Mining Twitter for suicide prevention. Natural Language Processing and Information Systems.

